# Anxiety, depression and perseverative cognition in women cycling naturally or taking oral contraceptives – a diary study

**DOI:** 10.1186/s40359-025-03437-x

**Published:** 2025-10-14

**Authors:** Melanie Kowalczyk, Monika Kornacka, Izabela Krejtz

**Affiliations:** 1 Institute of Psychology, SWPS University, Chodakowska 19/31, Warsaw, 03-815 Poland; 2 Institute of Psychology, SWPS University, Techników 9, 40-326, Katowice, Poland

**Keywords:** Anxiety, Depression, Menstrual cycle, Oral contraceptives, Daily diary, Perseverative cognition, Self-esteem, Satisfaction with life

## Abstract

**Background:**

While the literature is inconsistent about the link between anxiety and oral contraceptives (OC), the link between depression and OC is recognized in women who started OC during adolescence. Perseverative cognition is a stress-coping mechanism present both in anxiety and depression. Our study aimed to understand the differences between women taking OC and naturally cycling (NC) women regarding their daily levels of anxiety, depression, related negative factors (perseverative cognition and stress), and protective factors (self-esteem and life satisfaction).

**Methods:**

The study was conducted on 89 adult women (*M* age = 26.60, *SD* = 4.65) (48 – OC; 41 – NC) who participated in a 15-day online diary study divided into 3 phases throughout one menstrual cycle. The women using OC were further classified according to the androgenicity of their OC (androgenic and anti-androgenic). The participants were sent an email in the evening with questionnaires they had to complete on the same day. The daily measures were taken from existing trait-level scales and adapted for daily administration. The analyses were conducted using hierarchical linear modeling, with a significance level of *p* < 0.05.

**Results:**

There were no differences between groups in daily levels of anxiety, perseverative cognition, and stress. However, anti-androgenic OC users had higher levels of daily depression than NC women in the three menstrual cycle phases tested for, while androgenic OC users had higher levels of daily depression than NC women in two phases. Both groups of women taking OC had lower levels of daily self-esteem than NC women in all three phases. Androgenic OC users had higher daily satisfaction with life than anti-androgenic OC users in one testing phase.

**Conclusions:**

OC users had higher daily levels of depression and lower daily levels of self-esteem than NC women. Future studies could explore the link between depression and the different types of OC in adult women, especially with longitudinal methods.

## Background

### Menstrual cycle and oral contraceptives

The menstrual cycle of women who cycle naturally is composed of two distinct phases: first, a follicular phase, and then a luteal phase [1]. As described by Schmalenberger et al. [2], the beginning of the menstrual cycle corresponds with the onset of menstruation when the levels of estradiol and progesterone are low. In the middle of the follicular phase, estradiol levels increase until they reach a peak just before ovulation. The luteal phase begins after ovulation. Progesterone levels start to rise before reaching their peak during the mid-luteal phase. At the same time, estradiol remains at a moderate level with a slight peak in the middle of the phase. The menstrual cycle ends when a sharp decline in the levels of estradiol and progesterone triggers the next menstruation. The luteal phase (i.e., premenstrual phase) has been linked with an exacerbation of numerous disorders, including anxiety disorders and depression (for a review, see Pinkerton et al. [3]. Compared to naturally cycling (NC) women, oral contraceptive (OC) users exhibit significantly lower levels of estradiol and progesterone [4] and a lack of hormonal variability [4]. The composition of combined OC is an association between ethinylestradiol and progestins, with the progestins being either androgenic or anti-androgenic [5]. Androgenic progestins originate from testosterone, whereas anti-androgenic progestins block androgen receptors [6]. The literature reviews conducted by Laird et al. [7] and Beltz [8] on the link between OC, mental disorders, and cognitive functions demonstrated a lack of consistency in the results obtained in previous studies.

### Anxiety, the menstrual cycle, and oral contraceptives

Generalized anxiety disorder (GAD) affects 4.5% of the population worldwide [9], and women are twice as likely as men to suffer from it [10]. According to the Diagnostic and Statistical Manual of Mental Disorders, Fifth Edition (DSM-5) [11], some symptoms of GAD are excessive anxiety and worry and an inability to control them, associated with different symptoms such as irritability or muscle aches. Changes in anxiety symptoms are associated with the menstrual cycle, i.e., symptoms increase before menstruation and at the beginning of menstruation (for a systematic review, see Green & Graham [12]). The most consistent findings in the literature are retrospective self-reports of an increase in anxiety symptoms during the premenstrual phase (for a review, see Nillni et al. [13]).

The review by Laird et al. [7] pointed to a lack of studies on the link between GAD and OC use, and the only cited research found that OC users were less likely to suffer from GAD [14]. A randomized controlled trial (RCT) conducted by Lundin et al. [15] showed that adult women experienced an increase in anxiety after starting OC, compared to the placebo group, over 3 months of use. The lack of studies on the link between OC and anxiety symptoms and the lack of consensus in the literature indicate a need to conduct more studies on this topic.

### Depression, the menstrual cycle, and oral contraceptives

Similar to the higher prevalence of GAD in women, they are also twice as likely as men to suffer from depression [16]. The DSM-5 [11] indicates that depression is accompanied by symptoms such as a depressed mood, a loss of interest and pleasure, feelings of worthlessness, or decreased concentration. Women’s increased risk of suffering from depression starts with puberty and decreases after menopause [17]. Moreover, the premenstrual and menstrual phases are linked with an increase in depression (for a review, see Handy et al. [18]).

The only consensus found on the link between the use of OC and depression suggests that starting OC during adolescence is related to a vulnerability to receiving a diagnosis of depression in adulthood [19, 20]. Regarding the link between depression and the use of OC in adult women, results differ between studies. Some studies have not found any association between combined OC and depression [21], while others have found an increase in depressive symptoms in the first two years after starting OC [22]. The established link between OC use during adolescence and the increased risk of developing depression in adulthood indicates a need to delve deeper into the relationship between OC use and depression in adult women.

### Factors related to anxiety and depression

To obtain accurate results, we studied negative variables linked with anxiety and depression, namely perceived stress and perseverative cognition. Higher levels of perceived stress are linked with higher levels of anxiety [23], and stress plays a role in the onset of depression [24]. According to the literature, one of the key transdiagnostic factors being a common mechanism to anxiety, depression, and stress, might be perseverative cognition [25]. It has been defined as “the repeated or chronic activation of the cognitive representation of one or more psychological stressors” ( [26] p. 114). This stress-coping mechanism, present both in anxiety and depression [27, 28], has a higher prevalence in women [29]. Additionally, the literature shows that it can enclose patients in a vicious circle where perseverative cognition triggers psychopathology symptoms and the symptoms cause more rumination (typical for depression [30], or worry (typical for anxiety [31], , impairing also instrumental behavior and problem solving [30]. Our study explored separately those two types of perseverative cognition, which have rarely been studied when comparing OC users and NC women.

Among trait dispositions considered as a protective factor allowing one to better handle anxiety, positive psychology literature points to low self-esteem as a predictor of higher levels of anxiety and depression [32]. We also measured daily satisfaction with life, as GAD is associated with a higher likelihood of experiencing lower life satisfaction and lower well-being [33]. Lower levels of life satisfaction are also associated with a higher risk of developing depressive symptoms [34].

### Aim of the study

The goal of our study was to examine the differences between daily anxiety and depression levels between OC users and NC women, as well as the protective and negative factors associated with these symptoms, while taking into account the different types of OC (androgenic and anti-androgenic).

#### Hypothesis 1

There is a link between anxiety and depression, between anxiety and perseverative cognition, and between depression and perseverative cognition when measured daily [25, 27, 28].

#### Hypothesis 2

There are differences between NC women and OC users in daily levels of anxiety [14, 15] and depression [22], as well as in the daily levels of the protective (self-esteem and satisfaction with life) and negative factors (perseverative thinking, worry, stress) associated with these symptoms.

#### Hypothesis 3

NC women have higher daily levels of anxiety and depression in their luteal phase [3]. The daily levels of perseverative thinking, worry, and stress are higher in this phase, whereas the daily levels of self-esteem and satisfaction with life are lower.

#### Hypothesis 4

Since OC users do not experience fluctuations in their hormonal levels [4], they also do not experience significant fluctuation in their daily levels of anxiety and depression throughout their menstrual cycle. The daily levels of the protective and negative factors associated with these symptoms do not change significantly either.

## Methods

### Participants

For this study, 103 women volunteered to participate, but 14 of them dropped out before the end of the study or had their results withdrawn due to potential unreliability or irregularity in their menstrual cycles. Participants who did not take part in the three phases of the diary were also excluded. The final sample included 89 women (*M* age = 26.60, *SD* = 4.65) with 41 NC women and 48 taking OC. The groups did not significantly differ in terms of age (*t* [71] = 0.70, *p* = 0.71). The women using OC were further classified according to the androgenicity of their OC [7, 21]: 32 in the anti-androgenic group and 16 in the androgenic group.

The inclusion criteria for participants in the study were: being between 18 and 45 years of age, Polish speakers, taking OC or naturally cycling with a regular menstrual cycle (according to Fehring et al. [1], a regular menstrual cycle is of a duration between 21 and 35 days with less than 7 days of difference between cycles). To conduct the diary study, participants were additionally required to have cycles that lasted at least 28 days, so that the phases did not overlap. If the participants reported a regular menstrual cycle with a variability of 28 to 35 days, the mean length of their menstrual cycle was calculated. The exclusion criteria were based on Kowalczyk et al. [35], namely: “using other forms of hormonal contraception, using a copper intrauterine device, having undergone sterilization, having stopped taking OC in the past 6 months, being pregnant or breastfeeding” (p. 243). The descriptive statistics of participants are reported in Table 1.

**Table 1:** Descriptive statistics for participants

	General sample (*N* = 89)	A. OC (*n* = 16)	Anti-a. OC (*n* = 32)	NC (*n* = 41)
	*M (SD)*	*M (SD)*	*M (SD)*	*M (SD)*
Age	26.60(4.67)	27.88(3.93)	25.13(4.18)	27.24(5.08)
Cycle length	29.24(1.70)	28.38(0.89)	28.75(1.37)	29.95(1.90)

Note. A.: androgenic; Anti-a.: anti-androgenic

The sample size was chosen based on Nezlek’s [36] and Welz’s [37] recommendations. Nezlek [36] suggests that the ideal sample size for a two-week daily diary study is approximately 100 participants. Welz noted that, due to the high drop-out rate in diary studies, there should be more than 60 participants.

The participants received a gift voucher of around €50 for participating in this study. The study was conducted in compliance with the Helsinki Declaration, and the research protocol was approved by the Ethics Committee of our institution (Decision No. 55/2020/2).

### Materials

#### Questionnaires

The following questionnaires were used at the beginning of the study: the GAD-7 Scale [38], the Perseverative Thinking Questionnaire (PTQ [27], , the Penn State Worry Questionnaire (PSWQ [39], , the Perceived Stress Scale (PSS [40], , and the Self-Esteem Scale (SES [41], . The descriptive statistics for these responses are reported in Table 2. The reliability of the scales (Cronbach’s alpha) was acceptable. The groups did not significantly differ on the trait level of the tested variables before the implementation of the diary procedure (see Table 2).

**Table 2:** Descriptive statistics for pre-test measures by groups

Measure	Reliability (Cronbach’s alpha)	M (SD)			
		NC (*n* = 41)	OC (*n* = 48)	A. OC (*n* = 16)	Anti-a.OC (*n* = 32)
GAD-7	0.89	6.63(4.76)	6.67(3.94)	6.06(3.36)	6.97(4.22)
PTQ	0.93	32.93(12.76)	34.73(9.44)	36.50(6.63)	33.84(10.55)
PSWQ	0.92	54.29(14.13)	57.90(10.67)	55.25(10.98)	59.22(10.43)
PSS	0.87	20.05(7.44)	20.54(6.83)	21.63(7.45)	20(6.56)
SES	0.82	17.34(5.12)	15.79(5.45)	15.56(5.29)	15.91(5.61)

Note. A.: androgenic; Anti-a.: anti-androgenic; NC: naturally cycling; OC: oral contraceptives; GAD: generalized anxiety disorder; PTQ: Perseverative Thinking Questionnaire; PSWQ: Penn State Worry Questionnaire; PSS: Perceived Stress Scale; SES: Self-Esteem Scale

Most of the daily measures were taken from existing trait-level scales and adapted for daily administration. This procedure of item adaptation followed the guidelines discussed in Nezlek [36]. All daily items included the word “today” to maximize the likelihood that participants were describing how they felt each day. Participants provided answers ranging from 1 (“not at all”) to 7 (“very much”).

*Daily anxiety*. The daily measures for anxiety consisted of three items selected from the GAD-7 scale [38]. The main question was “How often today have you been bothered by the following problems?”. The items chosen from the GAD-7 were items 1 (“Feeling nervous, anxious or on edge”), 3 (“Worrying too much about different things”), and 7 (“Feeling afraid as if something awful might happen”).

*Daily depression*. The daily measures of depression consisted of three items corresponding to the elements of Beck’s cognitive triad [46], adapted by Nezlek & Gable [42]. The first item was related to the negative view of self (“Overall, how positively did you feel about yourself today?”), the second item to the negative view of life in general (“Thinking of your life in general, how well did things go today?”) and the third item to the negative view of the future (“How optimistic are you about how your life will be tomorrow?”). These items were then reverse-coded.

*Daily perseverative cognition (perseverative thinking and worry).* The daily measures for perseverative thinking were three items chosen from the Perseverative Thinking Questionnaire (PTQ; [27]). The main question was “How often today have you been bothered by the following problems?”. The items chosen from the PTQ were items 1 (“The same thoughts kept going through my mind again and again.”), 4 (“I think about many problems without solving any of them.”) and 10 (“My thoughts prevented me from focusing on other things.”). The questions measuring daily levels of worry were adapted from Thielsch et al. [48] for daily administration: “How much did you worry today?” and “How much were you bothered by worry today?”

*Daily stress*. The daily measures to assess stress levels consisted of three items from the Perceived Stress Scale (PSS [40], . The items chosen were items 2 (“In the last month, how often have you felt that you were unable to control the important things in your life?” which was transformed into “How often today did you feel that you were unable to control the important things in your life?”), 6 (“In the last month, how often have you found that you could not cope with all the things that you had to do?” which was transformed into “How often today did you feel that you could not cope with all the things that you had to do?”) and 10 (“In the last month, how often have you felt difficulties were piling up so high that you could not overcome them?” which was transformed into “How often today did you feel that difficulties were piling up so high that you could not overcome them?”).

*Daily self-esteem*. The daily measures for self-esteem were three items chosen from the Self-Esteem Scale (SES [41], . The main question was “How often today have you felt that:” and the items chosen from the questionnaires were items 1 (“On the whole, I am satisfied with myself” transformed into “How often today have you felt that on the whole, you were satisfied with yourself?”), 2 (“At times, I think I am no good at all” transformed into “How often today have you felt that you were no good at all?”), 3 (“I feel that I have a number of good qualities” transformed into “How often today have you felt that you had a number of good qualities?”). Item 2 was reverse-coded.

*Daily satisfaction with life*. To determine daily life satisfaction, three questions were asked. Two were based on Oishi et al. [43], as in Nezlek et al. [44]: “How was today?” with answers ranging from 1 (“terrible”) to 7 (“excellent”) and “How satisfied were you with your life today?” with answers from 1 (“very dissatisfied”) to 7 (“very satisfied”). The last question was based on item 4 of the Psychological Well-Being Scale [45]: “The demands of everyday life often get me down,” which was transformed into “How much did the demands of everyday life get you down today?”. The third item was rated from 1 (“not at all”) to 7 (“very much”) and was then reverse-coded.

#### Diaries

Each participant was provided with an individual menstrual cycle calendar, which was calculated based on the date of her last menstruation onset and the usual length of her menstrual cycle. The calendar was divided into three phases, which we defined as the menstrual, follicular, and luteal phases for the NC women. The menstrual phase is part of the follicular phase but corresponds to menstruation in our study. Three parallel phases were chosen for the OC users. In each phase, measures were taken for 5 consecutive days, totaling 15 days, as per Nezlek’s method [36]. The menstrual phase started on the first day of menstruation until the 5th day. The follicular phase was calculated based on the expected ovulation, which usually happens 14 days before the next menstruation [1]. In Hidalgo-Lopez et al. [46], the pre-ovulatory phase begins 3 days before ovulation; therefore, we calculated the follicular phase as spanning 5 days before the pre-ovulatory phase. Finally, the luteal phase is usually stable and lasts 14 days [1]. In Hidalgo-Lopez et al. [46], this phase was measured over a relatively long span (from 3 days post-ovulation to 3 days before the next menstruation); therefore, we based our calculations on Welz et al. [37], who tested participants on the 6th and 5th day before the next menstruation. As a result, the luteal phase in our study spans from the 9th to the 5th day before the next menstruation. For example, for a menstrual cycle of 28 days, the measured days were as follows: day 1–5 (menstrual), day 7–11 (follicular), and day 20–25 (luteal). The NC participants received commercially available ovulation tests (Horien Medical), which measure the luteinizing hormone (LH) surge in urine. These tests were conducted at home by the participants, who received an email at the end of the follicular phase, instructing them to test for ovulation for 10 consecutive days and to provide the experimenters with the date on which the test indicated an LH surge. If the ovulation test indicated a mismatch in the cycle phases, the dates of the phases were adjusted, and participants were given the opportunity to take part in the study again the following month. This offer was repeated for a second month, following which, if their menstrual cycle showed too much variability over the span of three cycles, their results were excluded from the study. OC users reported cycles that lasted around 28 days, and the three phases during which they were tested corresponded to the same days NC women were tested when they had menstrual cycles lasting 28 days. We did not separate the active and inactive pill phases as the OC users’ phases were meant to match the phases of the NC women to observe the results in the treatment group (OC) and the non-treatment group (NC) simultaneously throughout a whole menstrual cycle. The goal was to compare how NC women and OC users feel throughout their menstrual cycle and whether the lack of hormonal variability is linked with a difference in well-being. The first phase of OC users began on the first day of withdrawal bleeding, which happens during the inactive phase of OC [47]. OC users resumed their treatment during the 5 days corresponding to the first phase of their study (active phase). The second phase was tested during their active phase (days 7–11). We did not verify whether the OC users decided to observe a stop week at the end of their menstrual cycle. If they did not, then their third phase corresponded to the active phase. If they did, then based on the fact that they had a menstrual cycle of 28 days on average, their withdrawal bleeding started between 4 and 7 days after their last pill intake, depending on whether they were taking a 21 or 24-day OC. In this case, their last phase (days 20–25) represented a mixture of active and inactive phases. Precise information about the oral contraceptives taken by our participants can be found in Table 3.

**Table 3:** Oral contraceptives used by the participants

Oral contraceptive brands	Active and placebo	Active substance	Concentration µg/µg	*N*	Androgenic or anti-androgenic
Drovelis	24 + 4	Estetrol/drospirenone	14,200/3000	2	anti-androgenic
Zoely	24 + 4	Estradiol valerate/nomegestrol acetate	1500/2500	2	anti-androgenic
Belara	21	Ethinyl estradiol/chlormadinone acetate	30/2000	3	anti-androgenic
Novynette, Ovulastan	21	Ethinyl estradiol/desogestrel	20/150	2	androgenic
Aidee, Atywia, Jeanine	21	Ethinyl estradiol/dienogest	30/2000	8	anti-androgenic
Atywia Daily	21 + 7	Ethinyl estradiol/dienogest	30/2000	1	anti-androgenic
Axia Conti, Daylette, Vixpo	24 + 4	Ethinyl estradiol/drospirenone	20/3000	3	anti-androgenic
Teenia, Yasminelle	21	Ethinyl estradiol/drospirenone	20/3000	2	anti-androgenic
Vibin mini	21 + 7	Ethinyl estradiol/drospirenone	20/3000	5	anti-androgenic
Drosfemine Forte, Lesine,	21	Ethinyl estradiol/drospirenone	30/3000	2	anti-androgenic
Vibin	21 + 7	Ethinyl estradiol/drospirenone	30/3000	2	anti-androgenic
Kontracept	21	Ethinyl estradiol/gestodene	20/75	3	androgenic
Sylvie 30	21	Ethinyl estradiol/gestodene	30/75	1	androgenic
Vines	24 + 4	Ethinyl estradiol/gestodene	15/60	5	androgenic
Orlifique	21 + 7	Ethinyl estradiol/levonorgestrel	20/100	1	androgenic
Microgynon 21, Rigevidon	21	Ethinyl estradiol/levonorgestrel	30/150	2	androgenic
Elin	21	Ethinyl estradiol/norgestimate	35/250	2	androgenic
Slinda	24 + 4	Drospirenone	4000	2	anti-androgenic

### Procedure

The data were collected between February and December 2022. The participants were recruited online. Our institution shared an advertisement on social media. Interested candidates were presented with a description of the study and the required criteria to participate. They provided informed consent before taking part in the recruitment process. At the beginning of the study, before they began the diary, participants completed a set of individual difference measures on the Qualtrics platform [48], the results of which have been published previously [35]. Based on the diary study by Sztachańska et al. [49] and Nezlek’s method [36], all participants were sent an email at 8 pm during the 15 days of their menstrual cycle calendar with a link to the diary. They were asked to complete the daily questionnaire on that same day, and we accepted results that had been sent before 5 AM the next day. The results of participants who completed the diary later were not included.

### Statistical analysis

We conducted an exploratory diary study comparing NC women in three phases of their menstrual cycle (menses, follicular, and luteal) to women taking OC who were also tested three times, in parallel to the phases of the NC.

women. Our goal was to study parallel and dynamic changes in the participants’ daily levels of anxiety and depression. We wanted to explore whether the lack of hormonal fluctuation in OC users would be linked with higher or lower levels of these symptoms. Of particular importance was the comparison between the luteal phase of NC women and the phase leading up to menstruation in OC users, since NC women tend to experience an increase in negative symptoms before menstruation [12].

The analyses were conducted using hierarchical linear modeling. The data represents a two-level nested structure. Level 1 variables (data collected through diary measures) were nested in participants (Level 2). The number of diary entries collected for the 89 participants was *N* = 1178. The daily measures were separated into cycle phases (menstrual, follicular, luteal). Although OC users do not experience these phases in their menstrual cycle, we retained the same names for their phases to facilitate comparison with the phases of the NC group. Group types (anti-androgenic OC, androgenic OC, NC) were introduced at Level 2. The analyses followed the instructions provided by Nezlek [36] and were conducted using the HLM program [50]. Additionally, size effects were calculated using WebPower [51], and confidence intervals (CI) were calculated through *f*-square Effect Size Confidence Interval Calculator [52]. In the first part of the analysis, we ran an unconditional model for each of the Level 1 variables with no predictors at Level 1 or Level 2 to estimate sources of variability, whether the variability related to days (Level 1) or participants (Level 2):

1 $$\begin{array}{l}\text{L}\text{e}\text{v}\text{e}\text{l}\:1\:(\text{w}\text{i}\text{t}\text{h}\text{i}\text{n}-\text{p}\text{e}\text{r}\text{s}\text{o}\text{n}):\:Anxiet{y}_{ij}\:=\:{{\beta}}_{0j}\:+\:{r}_{ij}\\\text{L}\text{e}\text{v}\text{e}\text{l}\:2\:(\text{b}\text{e}\text{t}\text{w}\text{e}\text{e}\text{n}-\text{p}\text{e}\text{r}\text{s}\text{o}\text{n}):\:{{\beta}}_{0j}\:=\:{\gamma}_{00}\:+\:{u}_{0j}\end{array}$$

To test the link between daily anxiety and daily depression (hypothesis 1), we used the following equation:

2 $$\begin{array}{*{20}{l}}{{\rm{Level}}\:{\rm{1}}\:{\rm{(within - person):}}\:{\rm{Anxiet}}{{\rm{y}}_{ij}}\: = \:{\beta _{1j}}\:\left( {depression} \right)\: + \:{r_{ij}}}\\{{\rm{Level}}\:{\rm{2}}\:\left( {{\rm{between}}\:{\rm{person}}} \right):\:{\beta _{1j}}\: = \:{\gamma _{10}}}\end{array}$$

Similar equations were used to test the link between daily levels of anxiety and perseverative cognition, as well as daily levels of depression and perseverative cognition.

To test the differences between the groups on anxiety levels in the different cycle phases (hypothesis 2), we used the following equation:

3 $$\begin{array}{*{20}{l}}{\begin{array}{*{20}{l}}{{\rm{Level}}\:1\:\left( {{\rm{within}} - {\rm{person}}} \right):\:{\rm{Anxiet}}{{\rm{y}}_{ij}}\: = \:\beta {\:_{1j}}\:\left( {Menstrual} \right)}\\{ + \:\beta {\:_{2j}}\left( {Follicular} \right) + \:\beta {\:_{3j}}\left( {Luteal} \right) + \:{r_{ij}}}\end{array}}\\{\begin{array}{*{20}{l}}{{\rm{Level}}\:2\:\left( {{\rm{between}}\:{\rm{person}}} \right):\:}\\\begin{array}{l}{\beta _{1j}}\: = \:{\gamma _{10}}\:\left( {androgenic} \right) + \:{\gamma _{20}}\:\\\left( {anti - androgenic} \right) + \:{\gamma _{30}}\:\left( {NC} \right) + \:{u_{1j}}\end{array}\\\begin{array}{l}{\beta _{2j}}\: = \:{\gamma _{11}}\:\left( {androgenic} \right)\: + \:{\gamma _{21}}\:\\(anti - androgenic)\: + \:{\gamma _{31}}\:\left( {NC} \right)\: + \:{u_{2j}}\end{array}\\\begin{array}{l}{\beta _{3j}}\: = \:{\gamma _{12}}\:\left( {androgenic} \right)\: + \:{\gamma _{22}}\:\\(anti - androgenic)\: + \:{\gamma _{32}}\:\left( {NC} \right)\: + \:{u_{3j}}\end{array}\end{array}}\end{array} $$

The same equations were used for the other dependent variables.

To test whether there are between-phases differences in anxiety levels for the NC women (hypothesis 3), we used the following equation:

4 $$\begin{array}{l}{\rm{Level}}\:1\:\left( {{\rm{within}} - {\rm{person}}} \right):\:{\rm{Anxiet}}{{\rm{y}}_{ij}}\: = \:{\beta _{1j}}\:\left( {Menstrual} \right) + \\\:{\beta _{2j}}\left( {Follicular} \right) + \:{\beta _{3j}}\left( {Luteal} \right) + \:{r_{ij}}\\{\rm{Level}}\:2\:\left( {{\rm{between}}\:{\rm{person}}} \right):\\{\beta _{1j}}\: = \:{\gamma _{10}}\:\left( {NC} \right)\\{\beta _{2j}}\: = \:{\gamma _{11}}\:\left( {NC} \right)\\{\beta _{3j}}\: = \:{\gamma _{12}}\:\left( {NC} \right)\end{array}$$

An analogous equation was used to test for between-phase differences in all the variables. These equations were repeated for the two other groups, androgenic OC users and anti-androgenic OC users (at Level 2) (hypothesis 4).

## Results

To obtain the descriptive statistics of the diaries, we ran Eq. 1. They are presented in Table 4 with the reliability of the scales.

**Table 4:** Descriptive statistics of the diary study

		Level 2 variables (*N* = 89)			
Daily Variable	Reliability	*M*	Variances Between	Within	
Anxiety	0.87	8.73	6.68	12.49	
Depression	0.91	10.60	5.75	7.03	
Perseverative thinking	0.90	7.85	7.73	10.90	
Worry	0.87	5.98	3.63	6.88	
Stress	0.91	8.90	10.21	13.65	
Self-esteem	0.94	13.57	8.87	8	
Satisfaction with life	0.79	12.97	2.45	8.32	

To test the link between daily anxiety and daily depression within the sample, we ran Eq. 2. A significant link was found between these two variables (*β =* 0.81, *SE* = 0.01, *p* < 0.001). A similar equation was used to test the link between daily anxiety and daily perseverative cognition, as well as the link between daily depression and daily perseverative cognition. There was a significant link between daily levels of anxiety and perseverative thinking (*β* = 1.04, *SE* = 0.01, *p* < 0.001), and between daily levels of anxiety and worry (*β* = 1.40, *SE* = 0.01, *p* < 0.001). There was also a significant link between daily levels of depression and perseverative thinking (*β* = 1.14, *SE* = 0.01, *p* < 0.001), and between daily levels of depression and worry (*β* = 1.52, *SE* = 0.02, *p* < 0.001).

### Differences between groups in daily measures within each phase

To test the differences between the groups on all the variables in the different cycle phases, we used Eq. 3. Contrast analyses did not reveal any significant differences between groups in daily levels of anxiety (*p*-values ranged from 0.221 to > 0.500), perseverative thinking (*p*-values ranged from 0.107 to > 0.500), worry (*p*-values ranged from 0.089 to > 0.500), and stress (*p*-values ranged from 0.150 to > 0.500). Significant differences between groups were found in daily levels of depression, self-esteem, and satisfaction with life. Anti-androgenic OC users had higher levels of daily depression (*β* = 10.66, *SE* = 0.29; *β* = 11.63, *SE* = 0.29; *β* = 11.28, *SE* = 0.29) than NC women (*β* = 9.64, *SE* = 0.26; *β* = 10.19, *SE* = 0.27; *β* = 10.12, *SE* = 0.27) in the menstrual phase (*p* = 0.008, *f* = 0.39, 95% CI [0.01, 0.35]), the follicular phase (*p* < 0.001, *f* = 0.55, 95% CI [0.09, 0.61]) and the luteal phase (*p* = 0.004, *f* = 0.44, 95% CI [0.03, 0.42]), respectively. Androgenic OC users also had higher levels of daily depression (*β* = 10.67, *SE* = 0.41; *β* = 11.35, *SE* = 0.43) than NC women (*β* = 9.64, *SE* = 0.26; *β* = 10.19, *SE* = 0.27) in the menstrual phase (*p* = 0.031, *f* = 0.39, 95% CI [0.01, 0.35]) and the follicular phase (*p* = 0.021, *f* = 0.44, 95% CI [0.03, 0.42]), respectively. Both anti-androgenic OC users (*β* = 13.37, *SE* = 0.33, *β* = 12.77, *SE* = 0.33, *β* = 13.03, *SE* = 0.33) and androgenic OC users (*β* = 12.77, *SE =* 0.47, *β* = 12.20, *SE* = 0.50, *β* = 12.77, *SE* = 0.48) had lower levels of daily self-esteem than NC women (*β* = 14.51, *SE* = 0.30, *β* = 14.25, *SE* = 0.30, *β* = 14.38, *SE* = 0.31) in the menstrual phase (*p* = 0.010, *f* = 0.40, 95% CI [0.01, 0.36]; *p* = 0.002, *f* = 0.62, 95% CI [0.14, 0.75]), the follicular phase (*p* = 0.001, *f* = 0.52, 95% CI [0.07, 0.56]; *p* < 0.001, *f* = 0.73, 95% CI [0.24, 1.01]) and the luteal phase (*p* = 0.003, *f* = 0.48, 95% CI [0.05, 0.49]; *p* = 0.005, *f* = 0.57, 95% CI [0.11, 0.65]), respectively. Androgenic OC users had higher daily satisfaction with life (*β* = 13.59, *SE* = 0.39) than anti-androgenic OC users (*β* = 12.46, *SE* = 0.27) when they were tested in the third phase (*p* = 0.016, *f* = 0.39, 95% CI [0.01, 0.35]). The comparisons of the variables in all groups and all menstrual cycle phases are presented in Table 5.

**Table 5:** Comparison of daily variables (Level 1) based on groups and menstrual cycle phases

Daily measures	Menstrual			Follicular				Luteal		
	A. OC	Anti-a. OC	NC	A. OC		Anti-a. OC	NC	A. OC	Anti-a. OC	NC
**Anxiety**										
*Coeff.*	9.23	8.78	8.50	9.06		8.73	8.61	9.21	8.74	8.52
*SE*	0.51	0.36	0.32	0.54		0.36	0.33	0.52	0.36	0.33
**Depression**										
*Coeff.*	10.67*	10.66**	9.64	11.35*		11.64***	10.19	10.94	11.28**	10.12
*SE*	0.41	0.29	0.26	0.43		0.29	0.27	0.42	0.29	0.27
**Perseverative thinking**										
*Coeff.*	8.40	8.16	7.45	8.24		8.06	8.04	7.53	7.81	7.41
*SE*	0.50	0.35	0.32	0.53		0.36	0.33	0.51	0.36	0.33
**Worry**										
*Coeff.*	6.41	6.12	5.66	6.35		6.03	6.05	5.94	6.02	5.78
*SE*	0.38	0.27	0.24	0.40		0.27	0.24	0.39	0.27	0.25
**Stress**										
*Coeff.*	9.71	8.72	8.77	9.09		8.56	9.09	8.69	9.05	8.86
*SE*	0.57	0.40	0.36	0.60		0.40	0.37	0.58	0.41	0.37
**Self-esteem**										
*Coeff.*	12.77**	13.37*	14.51	12.20***		12.77***	14.25	12.77**	13.03**	14.38
*SE*	0.47	0.33	0.30	0.50		0.33	0.30	0.48	0.33	0.31
**Satisfaction with life**										
*Coeff.*	13.74	12.77	13.47	12.97		12.57	12.63	13.59	12.46*	13.16
*SE*	0.38	0.27	0.24	0.40		0.27	0.25	0.39	0.27	0.25

*Note.* Bold font indicates a significant difference between three groups within each menstrual cycle phase; *p < 0.05; ** *p* < 0.01; *** *p* < 0.001. A.: androgenic; Anti-a.: anti-androgenic; NC: naturally cycling; OC: oral contraceptives.

### Differences between phases for daily measures in each group

To clarify whether there is a group effect between phases, we used Eq. 4. There was no significant difference in daily levels for any of the variables tested in the study between the three phases of the menstrual cycle in NC women, with all *p*-values greater than 0.500. There was also no significant difference in daily levels for any of the variables between the three times during which the anti-androgenic OC users and the androgenic OC users were tested, with all *p*-values greater than 0.500.

## Discussion

Our study had the advantage of using an intensive repeated measures method and taking into account androgenic and anti-androgenic OC users, following the recommendations of Laird et al. [7] and Beltz [8], who stressed the need to consider different kinds of OC when conducting studies on mental health disorders to gather accurate and consistent results and the need for more intensive longitudinal studies as a means to observe individual differences.

Our first hypothesis was supported, as in our diary study, there was a significant link between anxiety and depression levels within the sample. There was also a significant link between anxiety and perseverative cognition, as well as depression and perseverative cognition. These links have been previously established in the literature [25, 27, 28] and confirmed in our study.

Our second hypothesis was partly supported as there were no differences between groups in daily levels of anxiety, worry, perseverative thinking, and perceived stress. However, there were differences between groups in daily levels of depression, self-esteem, and satisfaction with life. Anxiety and depressive disorders tend to be highly comorbid [53], so worsening symptoms of one disorder could be linked with worsening symptoms of the other one. As a result, we could have expected that significantly higher levels of depression in the OC user groups compared to the NC women would be linked with significantly higher levels of anxiety, but it is not the case in our sample. Anti-androgenic OC users had higher daily levels of depression than NC women in all the phases of their menstrual cycle. In comparison, androgenic OC users had higher daily levels of depression than NC women who were in their menstrual and follicular phases. Both groups of women taking OC had lower daily levels of self-esteem than NC women in all phases of the menstrual cycle. Androgenic OC users had higher levels of daily satisfaction with life than anti-androgenic OC users in two out of the three time points tested.

The lack of differences between OC users and NC women on daily levels of anxiety is in line with certain studies conducted previously [35, 54, 55], which used the GAD-7 scale [38] and the State-Trait Anxiety Inventory questionnaires (STAI-I, STAI-II) [56] to compare NC women with women taking androgenic and anti-androgenic OC. However, the RCT conducted by Lundin [15] showed that women who started taking an anti-androgenic OC experienced an increase in anxiety levels in the 3 months of treatment during which they were monitored. Our study was only observational and could not draw causal conclusions. Cheslak-Postava et al. [14] analyzed data drawn from the 1999–2004 American National Health and Nutrition Examination Survey and found contrary results. They compared NC women with OC users, without specifying the kind of progestins present in the OC and discovered that women taking OC had lower levels of anxiety than NC women. It is challenging to make a direct comparison between our results and those of Cheslak-Postava et al. [14] due to the lack of information on progestin properties. However, we know that anti-androgenic OC have been developed and prescribed more since 2010, in place of the androgenic OC that used to be prescribed previously [57]. We can hypothesize, based on the time when the data were collected, that the women surveyed in the study by Cheslak-Postava et al. [14] were taking androgenic rather than anti-androgenic OC. This means that the use of androgenic OC might be linked with lower levels of anxiety. However, there is still a lack of studies conducted on the link between anxiety and OC [7]. It is also possible that these studies exist, but they lead to non-significant results (no difference between NC women and OC users) and are therefore not published. The lack of studies on this topic and the mixed results present in the current literature do not allow us to have enough data to draw firm conclusions on the link between anxiety levels and OC use. As the review by Beltz [8] underscores, conducting RCTs in the field of OC is challenging; however, it would be beneficial to conduct more studies evaluating the levels of anxiety before and after treatment initiation, particularly in the long term.

Even though we did not find differences in daily anxiety levels, we observed differences in daily levels of depression, with OC users having higher daily levels of depression than NC women in all their menstrual cycle phases. The only exception was the lack of difference between androgenic OC users and NC women in their luteal phase. Our results are in line with the population-based cohort studies conducted by Johansson et al. [22] and Skovlund et al. [20], which showed higher levels of depression in adult OC users, both in androgenic and anti-androgenic ones. However, the higher levels of depression in OC users in these studies were only observed at the beginning of the treatment. We did not ask our participants how long they had been taking OC, which does not allow us to know whether their higher levels of depression could have been due to being at the beginning of their treatment. Nevertheless, the literature remains contradictory regarding the link between OC use and depression levels in adult women. On the one hand, the systematic review and network meta-analysis of randomized clinical trials conducted by de Wit et al. [58] does not indicate a link between the use of OC in adult women and an increase in depressive symptoms. Some studies even show reduced levels of depression in OC users compared to NC women [59, 60]. These studies used the Center for Epidemiologic Studies Depression Scale (CES-D [61], and the Beck Depression Inventory-13 (BDI-13 [62], to assess depression, but did not control for the androgenicity of the OC. Nevertheless, one of these studies [59] underlines that women with a history of depression are less likely to choose hormonal contraception as a form of contraception in the first place. In contrast to the studies showing no difference between OC users and NC women, or lower levels of depression in OC users, a meta-analysis conducted by Pérez-López et al. [63] showed a link between hormonal contraceptive use and consumed suicides, without indicating the androgenicity of the OC. This information is important to take into account when studying the link between OC and depression, as the review by Sundström Poromaa and Segebladh [64] indicated that anti-androgenic OC are more efficient at lessening mood symptoms than androgenic OC. However, in our study, anti-androgenic OC users had higher levels of daily depression than NC women during the three phases, whereas androgenic OC users only had higher levels of daily depression than NC women in two phases (menstrual and follicular), but not in the luteal phase. This could be explained by the fact that androgenic OC users have lower levels of testosterone than NC women [65], but their levels of testosterone are not as low as those of anti-androgenic OC users [6], while NC women see a decrease in testosterone levels in the luteal phase [66]. Since testosterone has an antidepressant effect on men and women alike [67], it is possible that the depression levels of androgenic OC users do not differ from those of NC women in their luteal phase because their testosterone levels are more similar than those of anti-androgenic OC users and NC women.

We did not find any difference in the daily negative factors associated with anxiety and depression, namely worry, perseverative thinking, and perceived stress, between the NC women and the OC group. Few studies have compared these variables in NC women and OC users, let al.one using repeated measures. As a result, there is a lack of studies on these topics, and the results are contradictory. A study conducted by Kowalczyk et al. [35] concluded that women taking anti-androgenic OC had significantly higher levels of worry than NC women. In contrast, the study by Louis et al. [68] did not find any differences in worry levels between NC women and hormonal contraceptive users. OC users have also been shown to experience higher levels of neutral mind wandering, which is related to depressive symptoms [69]. Regarding stress levels, studies have shown that OC users have higher levels of cortisol [70] and a blunted cortisol response to stress [71] compared to NC women, which might indicate a state of chronic stress in OC users. However, our study did not find any difference between groups. The absence of a difference in daily stress levels between the OC group and the NC group allows us to conclude that the everyday life stress the participants might have encountered throughout the study could not have affected possible group effects. However, these results should be considered with some precautions, particularly that all variables were measured on a daily basis. Future studies may also explore within-day fluctuations (e.g [72]. , .

We found lower daily levels of self-esteem in both groups of OC users compared to NC women, suggesting, in line with the previous studies, that self-esteem is a protective factor against depression [32]. Another protective factor against depression is satisfaction with life, as lower levels of life satisfaction are associated with a higher risk of developing depressive symptoms [34]. We found that androgenic OC users had higher levels of daily satisfaction with life than anti-androgenic OC users, but this result should be approached with caution, as it only appeared in one of the testing phases. To the best of our knowledge, no studies have investigated daily self-esteem or daily satisfaction with life in OC users before this. However, studies on the link between OC and general well-being have shown strongly heterogeneous results: hormonal contraceptive users showed lower levels of well-being than NC women in one study [73], androgenic OC users enrolled in an RCT had lower levels of well-being than NC women [74], another study concluded that anti-androgenic OC use was associated with a higher level of psychological well-being [75], while an RCT comparing androgenic and anti-androgenic OC use found an increase in emotional well-being in both groups during the treatment [76]. The heterogeneity of the results reported in the literature indicates an important need to conduct more studies on the link between OC use, well-being, self-esteem, and satisfaction in life, as these variables represent protective factors lowering the risk of developing an anxiety disorder or depression.

The third hypothesis was not supported, as no differences were observed in daily levels for any of the variables tested during the study between the different phases of the menstrual cycle in NC women. The literature indicates that there is an exacerbation of anxiety [3, 12, 13] and depression [3, 18] symptoms in the luteal phase of NC women, which is not the case in our sample. The lack of exacerbation of symptoms during the luteal phase in our study might be explained by the fact that our sample was not from a clinical population, as we did not know whether our participants had received an official diagnosis of GAD or depression. Moreover, the review by Kuehner and Nayman [77] concluded that around 60% of women suffering from depression experience an increase in their symptoms during the premenstrual phase, but that these conclusions are limited by retrospective assessments, no control of the presence of hormonal contraceptives and psychotropic drug use, and no reliable validation of ovulation. Our study was not retrospective; we verified oral contraceptive use and ovulation date, which might explain why we found different results. Our fourth hypothesis, however, was supported, as neither androgenic OC users nor anti-androgenic OC users showed any differences in any of the variables across the three time points. This finding is consistent with the literature, which has demonstrated that OC users tend to experience less variability in affect during their menstrual cycle compared to NC women [78–80].

### Limitations

While the present study addressed certain limitations from previous studies, such as separating androgenic and anti-androgenic OC or using intensive repeated measures, it is not free from its own limitations. For instance, we calculated the cycle phases based on the date of the participants’ last menstruation onset and the usual length of their menstrual cycle, and we also asked them to test for the date of their ovulation. However, we did not conduct any hormonal levels testing to confirm the reliability of the cycle phases. Our study was observational, and we cannot draw causal conclusions from it. We did not ask participants about previous use of OC and the reason for their treatment. For example, women with polycystic ovary syndrome show higher levels of androgenicity and are often prescribed anti-androgenic OC for their symptoms [81]. This could have contributed to a possible selection bias confound related to the groups. We did not compare the active and inactive phases of the OC users, and we did not ask the participants whether they were stopping their treatment during the last phase of the study, to experience withdrawal bleeding. We also did not compare the dose of ethinylestradiol present in the OC, as suggested by Beltz [8]. The perimenopausal status of the participants was not verified, and we also did not verify at what age the OC users had started their treatment. We did not ask whether the participants were taking any psychotropic medication or whether they had any comorbidities. Finally, while the number of participants was similar in the NC group and the OC group, the number of participants taking androgenic OC (*n* = 16) was much smaller than the number of participants taking anti-androgenic OC (*n* = 32).

### Future studies

The lack of consistency in the literature related to the link between OC use and anxiety and depression indicates a necessity to conduct more studies on these topics. Future studies might also take into account the link between testosterone and mental health issues in NC women and OC users, since it has been demonstrated that higher levels of testosterone are linked with lower levels of anxiety and depression [67]. Moreover, the differences between androgenic and anti-androgenic OC users in our study underline the need to control the kind of OC women are using. The review by Beltz [8] provides numerous indications on how to conduct future studies more reliably by taking into account various variables, such as assessing the exact phases of the menstrual cycle using hormonal measures, comparing the active and inactive phases of OC, or considering the composition of OC. Other limitations described in the review are a lack of longitudinal studies, especially those comparing women before and after they started taking OC, and small sample sizes. The present study has shown that protective variables related to handling anxiety and depression better (such as self-esteem or satisfaction with life) should be considered when studying those topics. It might also be important to control for other variables that could help refine the results, such as genetic predisposition (anxiety: [82]; depression: [83]), lifestyle factors [84], body mass index [85], menarche age [86, 87], or socioeconomic level [88]. It could be interesting to examine whether positive psychology interventions, which are efficient in alleviating symptoms of depression (meta-analysis: [89]), could help women who are taking OC and experiencing elevated levels of depressive symptoms.

## Conclusions

We believe that our study has contributed to the literature by conducting an intensive repeated measures assessment of anxiety and depression on a daily level and comparing NC women and OC users during multiple phases of the menstrual cycle while taking into account the differences between androgenic and anti-androgenic OC. We have demonstrated that OC use is linked with a higher level of daily depression and a lower level of daily self-esteem compared to natural cycling conditions.

## Data Availability

The datasets analyzed during the current study are available from the corresponding author upon reasonable request.

## Abbreviations

A.: Androgenic
Anti–a.: Anti–androgenic
GAD: Generalized anxiety disorder
LH: Luteinizing hormone
OC: Oral contraceptives
NC: Naturally cycling
RCT: Randomized controlled trial
